# Antiviral Activity of a Novel Compound CW-33 against Japanese Encephalitis Virus through Inhibiting Intracellular Calcium Overload

**DOI:** 10.3390/ijms17091386

**Published:** 2016-08-24

**Authors:** Su-Hua Huang, Jin-Cherng Lien, Chao-Jung Chen, Yu-Ching Liu, Ching-Ying Wang, Chia-Fong Ping, Yu-Fong Lin, An-Cheng Huang, Cheng-Wen Lin

**Affiliations:** 1 Department of Biotechnology, Asia University, Wufeng, Taichung 413, Taiwan; shhuang@asia.edu.tw; 2 School of Pharmacy, China Medical University, Taichung 404, Taiwan; jclien@mail.cmu.edu.tw; 3 Proteomics Core Laboratory, Department of Medical Research, China Medical University Hospital, Taichung 404, Taiwan; cjchen@mail.cmu.edu.tw (C.-J.C.); yuhchyan1104@yahoo.com.tw (Y.-C.L.); 4 Department of Medical Laboratory Science and Biotechnology, China Medical University, Taichung 404, Taiwan; spirit1126@hotmail.com (C.-Y.W.); anniepyng@hotmail.com (C.-F.P.); wnedy55@yahoo.com.tw (Y.-F.L.); 5 Department of Nursing, St. Mary’s Junior College of Medicine, Nursing and Management, Yilan County 266, Taiwan

**Keywords:** Japanese encephalitis virus, furoquinolines, Ca^2+^ overload, Akt/mTOR, Jak/STAT1

## Abstract

Japanese encephalitis virus (JEV), a mosquito-borne flavivirus, has five genotypes (I, II, III, IV, and V). JEV genotype I circulates widely in some Asian countries. However, current JEV vaccines based on genotype III strains show low neutralizing capacities against genotype I variants. In addition, JE has no specific treatment, except a few supportive treatments. Compound CW-33, an intermediate synthesized derivative of furoquinolines, was investigated for its antiviral activities against JEV in this study. CW-33 exhibited the less cytotoxicity to Syrian baby hamster kidney (BHK-21) and human medulloblastoma (TE761) cells. CW-33 dose-dependently reduced the cytopathic effect and apoptosis of JEV-infected cells. Supernatant virus yield assay pinpointed CW-33 as having potential anti-JEV activity with IC_50_ values ranging from 12.7 to 38.5 μM. Time**-**of**-**addition assay with CW-33 indicated that simultaneous and post-treatment had no plaque reduction activity, but continuous and simultaneous treatments proved to have highly effective antiviral activity, with IC_50_ values of 32.7 and 48.5 μM, respectively. CW-33 significantly moderated JEV-triggered Ca^2+^ overload, which correlated with the recovery of mitochondria membrane potential as well as the activation of Akt/mTOR and Jak/STAT1 signals in treated infected cells. Phosphopeptide profiling by LC-MS/MS revealed that CW-33 upregulated proteins from the enzyme modulator category, such as protein phosphatase inhibitor 2 (I-2), Rho GTPase-activating protein 35, ARF GTPase-activating protein GIT2, and putative 3-phosphoinositide-dependent protein kinase 2. These enzyme modulators identified were associated with the activation of Akt/mTOR and Jak/STAT1 signals. Meanwhile, I-2 treatment substantially inhibited the apoptosis of JEV-infected cells. The results demonstrated that CW-33 exhibited a significant potential in the development of anti-JEV agents.

## 1. Introduction

The *Flaviviridae* family contains a positive-sense RNA genome that encodes three structural proteins (capsid (C), membrane (prM/M), and envelope (E)) and seven non-structural proteins (NS1, NS2A, NS2B, NS3, NS4A, NS4B, and NS5). Mosquito-borne falviviruses, including Japanese encephalitis virus (JEV), dengue virus, West Nile virus, and Zika virus, have caused significant epidemic outbreaks in recent decades [1,2]. Over 3 billion people in East and Southeast Asia, along with northern Australia, are at risk of JEV infection; 30,000 to 50,000 JE cases, with 10,000 deaths and half of survivors showing severe neurological sequelae, are reported annually in these areas [3]. JEV causes a range of severe central nervous system disorders: acute flaccid paralysis, aseptic meningitis, and encephalitis. According to the nucleotide sequence of E protein, JEV variants are classified into five genotypes (I, II, III, IV, and V) [4]. Co-circulation of JEV genotypes occurs in the epidemic area, but genotype III has been predominant since the 1950s. Two vaccines of JEV genotype III, live attenuated vaccine SA14-14-2 strain and inactivated mouse brain vaccine Nakayama strain, have been used worldwide for vaccination against JEV infection [4]. Recently, the isolation of JEV genotype I from the specimens of mosquitoes, swine, and humans has significantly increased in Vietnam, Taiwan, China, and Korea, suggesting that JEV genotype I replaces genotype III as the predominant genotype in these countries [5,6,7]. Importantly, the neutralizing capacity of sera from children who received the inactivated JEV genotype III vaccine Nakayama strain is low against JEV genotype I [8], which was noticed when a JEV outbreak occurred in 2010 in one of these vaccination program countries [9]. The discovery of effective agents against JEV infection has become a global health issue.

**Figure 1:**
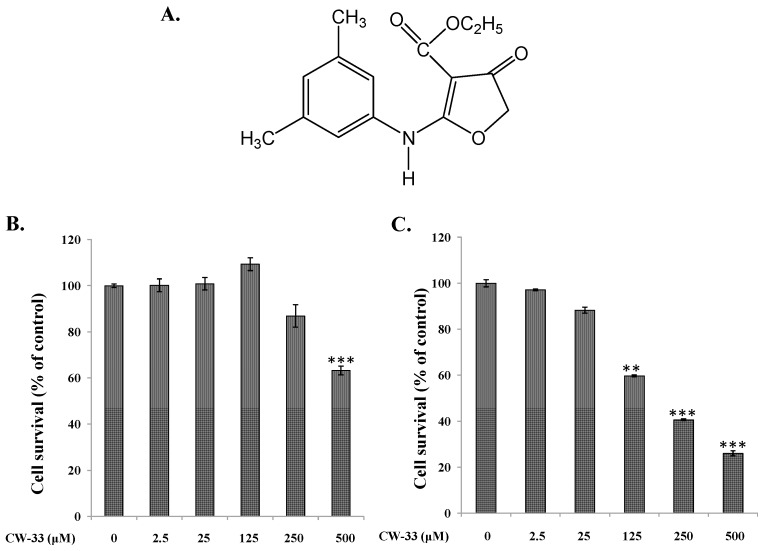
Survival rate of BHK-21 and TE671 cells in response to CW-33. The structure of CW-33 (Ethyl 2-(3′,5′-dimethylanilino)-4-oxo-4,5-dihydrofuran-3-carboxylate) is shown in (**A**). For antiproliferative assay, BHK-21 (**B**) and TE671 (**C**) cells cultured on 96-well plates were treated with CW-33, then incubated for 48 h, followed by MTT assay. Survival rates of cells were calculated as the ratio of OD_570–630_ of treated cells to OD_570–630_ of untreated cells. ** *p* value < 0.01; *** *p* value < 0.001 compared with mock-treated cells.

Furoquinoline alkaloids show anti-inflammatory [10,11], antifungal [12], antimicrobial [13], and anticancer activity [14]. A novel compound CW-33 (ethyl 2-(3′,5′-dimethylanilino)-4-oxo-4,5-dihydrofuran-3-carboxylate), a synthetic derivative of furoquinoline alkaloid, has been demonstrated to inhibit the replication of enterovirus A71 in vitro via the inhibition of viral 2A protease activity and the recovery of IFNAR1 protein levels [15]. Combined treatment with CW-33 and IFN-β exhibits a synergistic antiviral activity against enterovirus A71. In addition, the furanonaphthoquinone derivative 2-methylnaphtho [2,3-*b*]furan-4,9-dione (FNQ3) shows antiviral activity against JEV through the inhibition of viral RNA and protein synthesis [16]. Thus, this study intends to investigate the antiviral activity of a novel compound, CW-33 (Figure 1A), against JEV in vitro. CW-33 reduced the JEV-induced cytopathic effect and apoptosis, and also inhibited virus yield and plaque formation in a concentration-dependent manner. Importantly, CW-33 treatment reduced intracellular Ca^2+^ levels, raised mitochondrial membrane potential, and upregulated signal pathways of AKT/mTOR and JAK/STAT in JEV-infected cells. In addition, CW-33 activated the expression of IFN-stimulated genes (ISGs). Results demonstrated CW-33 exhibiting significant potential against JEV.

## 2. Results

### 2.1. Antiviral Activity of CW-33 against JEV (Japanese Encephalitis Virus)

**Figure 2:**
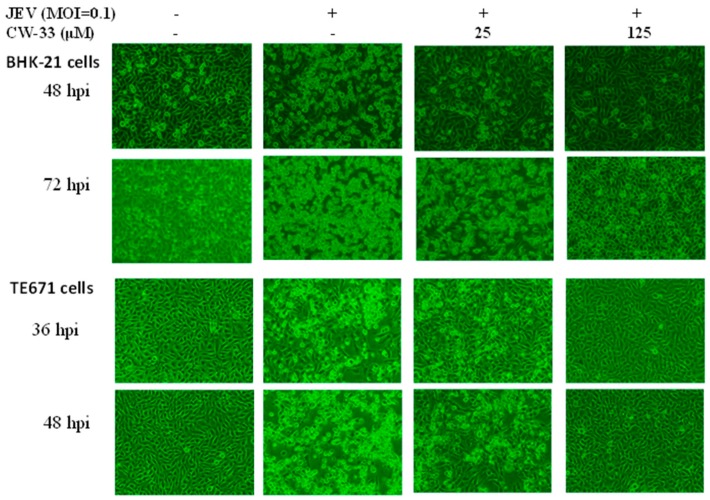
Reduction of JEV-induced cytopathic effects by CW-33. BHK-21 and TE671 cells were infected with JEV and immediately treated with CW-33. Virus-induced cytopathic effect was photographed 36, 48, or 72 h post-infection by phase-contrast microscopy.

**Figure 3:**
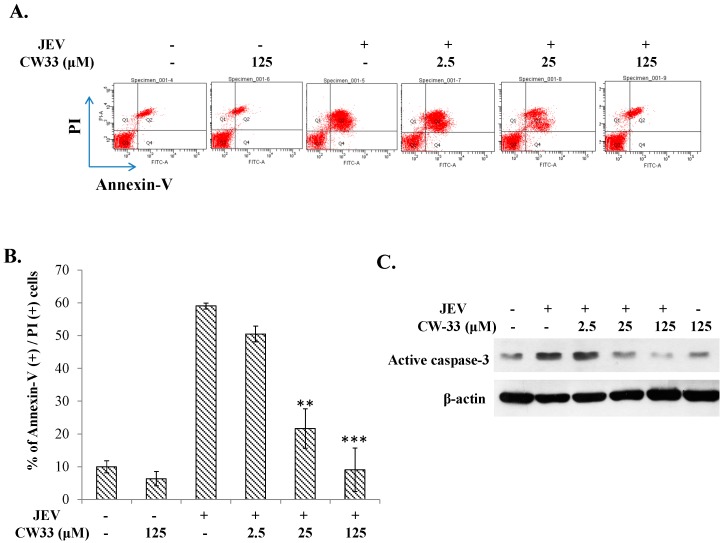
Inhibition of JEV-induced apoptosis by CW-33. TE671 cells were infected with JEV at a MOI of 0.1, and immediately treated with CW-33. Cells were harvested 36 h post-treatment, stained by Annexin V-FITC/PI dye, and then analyzed using flow cytometry (**A**); annexin V positive/PI positive presented as late apoptosis (**B**); active forms of caspases 3 in treated infected cells were characterized using Western blotting (**C**). ** *p* value < 0.01; *** *p* value < 0.001 compared with mock-treated infected cells.

**Figure 4:**
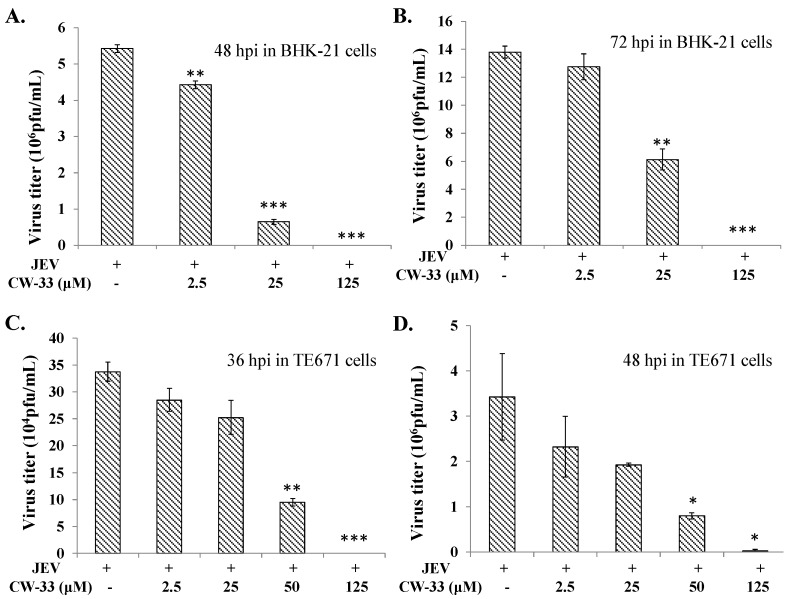
Virus yield reduction by CW-33. BHK-21 (**A**,**B**) and TE671 (**C**,**D**) cells were infected with JEV and immediately treated with indicated CW-33 concentration. Supernatant was harvested 36, 48, or 72 h post-infection, virus yield measured by plaque assay. * *p* value < 0.05; ** *p* value < 0.01; *** *p* value < 0.001 compared with mock-treated infected cells.

Initially, cytotoxicity of CW-33 was evaluated using MTT assays (Figure 1B,C). The 50% cytotoxicity concentration (CC50) values of CW-33 were greater 500 μM to BHK-21 cells and 189 μM to TE671 cells. Inhibitory assays of cytopathic effect (CPE) and apoptosis were subsequently used for determining the in vitro antiviral activity of CW-33 against JEV (Figure 2 and Figure 3). JEV-infected cells were treated with or without the various concentrations of CW-33 (2.5, 25 and 125 μM). Microscopic photography indicated CW-33 concentration-dependently reducing the JEV-induced cytopathic effect of BHK-21 cells 48 and 72 h post-infection, as well as TE671 cells 36 and 48 h post-infection, respectively (Figure 2). Annexin-V/PI apoptosis analysis using flow cytometry showed CW-33 significantly decreasing early and late apoptosis of JEV-infected cells in a dose-dependent manner (Figure 3A,B). Western blotting also showed CW-33 diminishing active form of caspase-3 in JEV-infected cells (Figure 3C). Supernatant virus yield using the plaque assay demonstrated CW-33 markedly inhibiting JEV production in BHK-21 and TE671 cells (Figure 4). The 50% inhibitory concentration (IC_50_) of CW-33 on inhibiting supernatant virus yield ranged from 12.7 to 38.5 μM based on the cell type and the harvest time. Notably, the therapeutic index value (CC50/IC_50_) of CW-33 against JEV exceeded 5. The results revealed the antiviral potential of CW-33 against JEV.

### 2.2. No Virucidal Activity and Attachment Inhibition by CW-33

**Figure 5:**
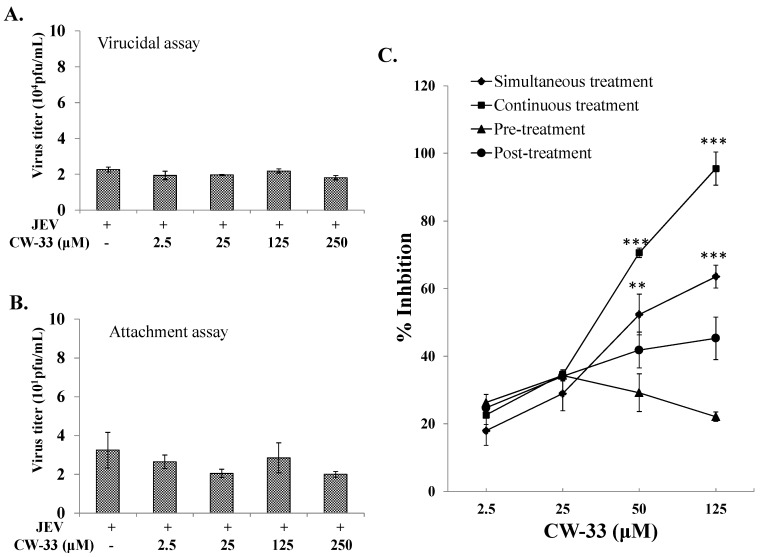
Modes of antiviral action of CW-33 against JEV. In a virucidal assay (**A**), JEV (10^6^ pfu) was mixed with CW-33, then incubated at 4 °C for 1 h; 1000-fold dilution of the JEV/CW mixture was used to determine the r*es*idual infectivity using plaque assay. In the attachment assay (**B**), JEV (50 pfu) was mixed with CW-33, then immediately added onto the BHK-21 cell monolayer for 1 h at 4 °C. After washing twice with PBS, monolayer was overlaid with 2 mL of a methylcellulose medium for three days at 37 °C in a CO_2_ incubator. Attachment inhibition was calculated as residual plaques. In time-of-addition assay (**C**), infected cells were treated with CW-33 1 h prior (pre), simultaneous, or 1 h post-infection and washed with PBS 1 h after treatment; infected cells were also treated with CW-33 in a continuous mode without the washing step, and followed by plaque assay. Inhibitory ratio was calculated from ratio of experimental data to mock-treated control. ** *p* value < 0.01; *** *p* value < 0.001 compared with mock-treated infected cells.

To ascertain whether CW-33 directly acts on JEV particles, virucidal activity assays were examined (Figure 5A). In virucidal activity assays, JEV was pre-incubated with CW-33 at 4 °C for 1 h and the 1000-fold dilution of each mixture was added on to the cell monolayer for quantifying their residual infectivity by plaque assay (Figure 5A). CW-33 exhibited no significant inhibitory effect on residual infectivity compared to controls. Results revealed CW-33 did not directly damage JEV particles. Examining the effect of CW-33 on virus attachment was further performed using viral attachment assay (Figure 5B). JEV was mixed with various concentrations of CW-33, and then immediately added onto the BHK-21 cell monolayer. After incubating at 4 °C for allowing attachment alone, each mixture was removed and the cell monolayer washed with PBS; residual infectivity was determined using plaque assay. The result of viral attachment assay indicated that CW-33 exhibited no significant effect on virus attachment (Figure 5B). A JEV replicon-expressing EGFP system further confirmed that CW-33 did not directly interfere with the infectivity of virus particles and early entry into cells. CW-33 significantly inhibited the transcription and translation during the JEV replication cycle, which reduced the viral RNA genome and proteins in a concentration-dependent manner (Supplementary Figures S1 and S2).

### 2.3. Time-of-Addition Effect of CW-33 against JEV

Antiviral action of CW-33 on JEV replication was further evaluated using time-of-addition assays including (1) prior (1 h before infection, and then washed out); (2) simultaneous (at the same time as infection for 1 h, and then washed out); (3) continuous (during the infection period); and (4) post (1 h after entry, 1 h incubation, and then washed out) treatment experiments (Figure 5C). Prior treatment of CW-33 had little effect on antiviral activity, implying that the prior treatment responses induced by CW-33 had no significance in suppressing JEV infection. By contrast, the simultaneous, continuous, and post-treatment experiments demonstrated CW-33 concentration-dependently reducing JEV plaques in vitro (Figure 5C). Among time-of-addition assays, continuous and simultaneous treatments with CW-33 were highly effective in terms of antiviral activity, with IC_50_ values of 32.7 and 48.5 μM, respectively. Results demonstrated that CW-33 had an inhibitory effect on late stages of JEV replication cycle.

### 2.4. Reduction of JEV-Induced Calcium Overload by CW-33

**Figure 6:**
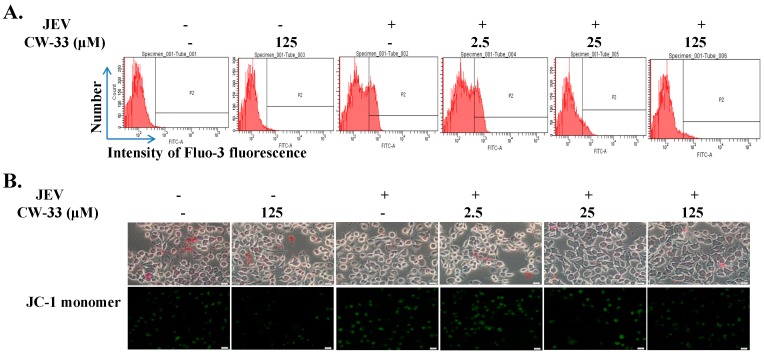
Effect of CW-33 on intracellular Ca^2+^ and mitochondrial membrane potential (ΔΨM) in JEV-infected cells. Infected cells were treated with CW-33 for 36 h, harvested and stained using Fluo-3/AM, and then analyzed by flow cytometry with excitation and emission spectra of 506 and 526 nm, respectively (**A**). Cells were also stained using a JC-1 dye, and then the green fluorescence of JC-1 monomer in the cells was photographed using a fluorescent microscope (**B**), scale bars = 50 µm.

Since calcium overload could be involved in the regulation of apoptosis via mitochondrial damage to activate caspase cascade [17,18,19], analysis of intracellular Ca^2+^ accumulation and mitochondria membrane potential (MMP) was performed using flow cytometry (Figure 6A) and JC-1 staining (Figure 6B). Flow cytometric analysis of intracellular Ca^2+^ with the dye FLUO3/AM revealed JEV causing the release of ER Ca^2+^ into cytosol, as well as CW-33 decreasing the cytosolic Ca^2+^ concentration in both cell types infected with JEV (Figure 6A). Similarly, JC-1 staining indicated that JEV infection triggered the green fluorescence of JC-1 monomer increase in cells as low MMP, while CW-33 significantly reduced the green fluorescence of JC-1 monomer in infected cells, indicating the increase of MMP (Figure 6B). Results revealed CW-33 reducing JEV-induced calcium overload, and then recovering the MMP of infected cells, which corrected the inhibitory effect of CW-33 on JEV-induced apoptosis.

### 2.5. Activation of Akt/mTOR and Jak/STAT Pathway in JEV-Infected Cells by CW-33

**Figure 7:**
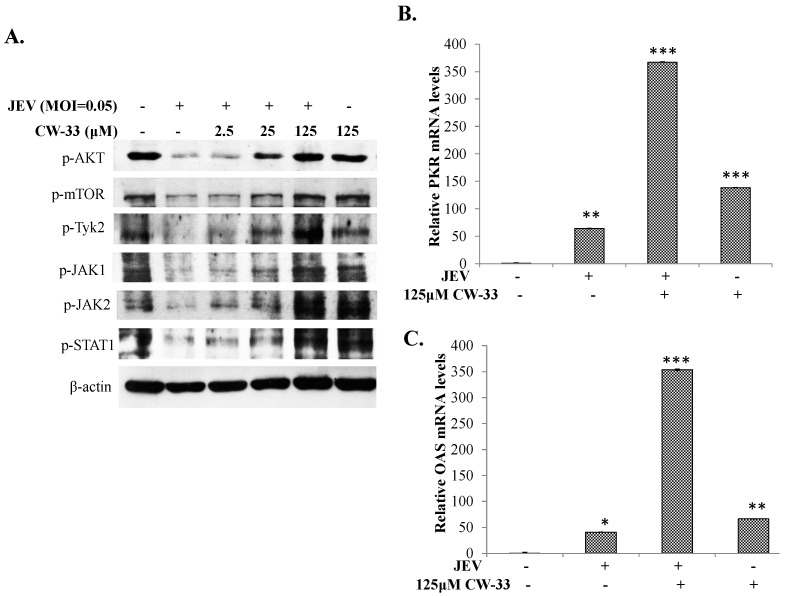
Activation of Akt/mTOR and Jak/STAT1 signaling in infected cells treated with CW-33. For assessing the phosphorylation of Akt, mTOR, Tyk2, Jak1, Jak2, and STAT1, lysates were separated on SDS-PAGE and transferred onto nitrocellulose paper. Blot was probed with specific mAbs, developed with enhanced chemiluminescence substrates (**A**). Relative mRNA expression of STAT1-dependent genes PKR (**B**) and 2′-5′ OAS (**C**) was measured with RT-PCR 36 h post-infection and treatment, and then normalized by housekeeping gene GAPDH. * *p* value < 0.05; ** *p* value < 0.01; *** *p* value < 0.001 compared with mock-treated cells.

The serine/threonine kinase Akt, an inhibitor of apoptosis, reduces the Ca^2+^ release from the Endoplasmic Reticulum to the mitochondria of apoptotic cells [20], thus the activity of Akt/mTOR in JEV-infected cells in the presence or absence of CW-33 was further examined using Western Blotting (Figure 7A). Interestingly, CW-33 raised the phosphorylation of Akt and mTOR suppressed by JEV in concentration-dependent manners (Figure 7A). In addition, the other survival pathways, ERK and Jak/STAT, were analyzed. CW-33 dose-dependently induced the phosphorylation of Jaks (Tyk2, Jak1, and Jak2) and STAT1 in JEV-infected cells (Figure 7A). The effect of CW-33 on the expression of STAT1-mediated genes in infected cells was subsequently assessed using real-time RT-PCR (Figure 7B,C). After normalization to the GAPDH, CW-33 alone induced upregulation of PKR and 2′,5′-OAS mRNA in mock cells by over 50-fold compared to mock control cells, whereas JEV infection also triggered the expression of both genes to be lower than that in cells treated with CW-33 alone. Importantly, CW-33 treatment caused a 300-fold higher increase of PKR and 2′-5′-OAS mRNA in JEV-infected cells. Patterns of PKR and 2′-5′-OAS mRNA levels in response to CW-33 and JEV infection correlated with activation of JAK/STAT signaling as the data of Western blotting (Figure 7A). Results revealed CW-33 reducing JEV-induced intracellular Ca^2+^, and activating Akt/mTOR, and Jak/STAT signaling pathways in infected cells.

### 2.6. Regulation of Protein Phosphatase-1 Inhibitor-2 (I-2) in JEV-Infected Cells by CW-33

**Figure 8:**
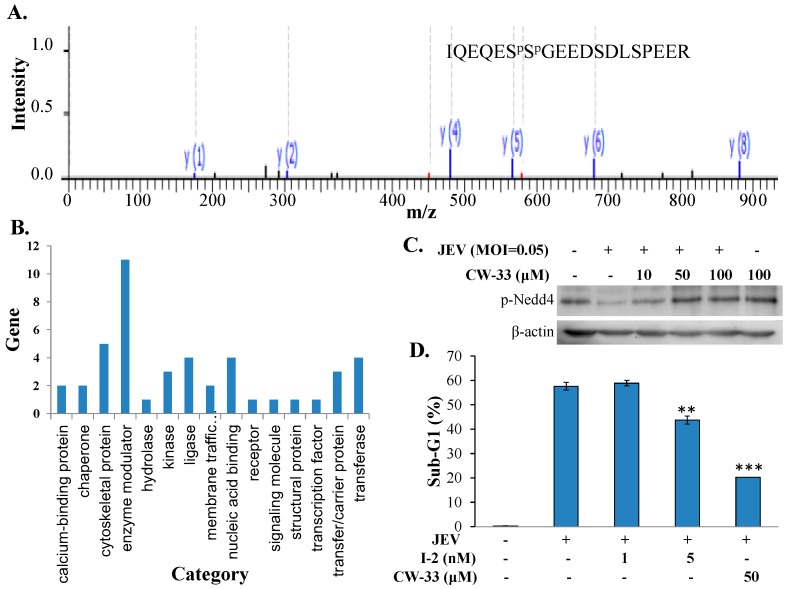
Phosphoproteome profiling of JEV-infected cells in response to CW-33. MS/MS spectrum of the phosphopeptide IQEQES^p^S^p^GEEDSDLSPEER matched in protein phosphatase 1 inhibitor 2 (I-2) is shown (**A**); regulated proteins identified in infected cells in response to CW-33 were categorized using PANTHER classification system (**B**); western blot analysis of lysates from treated and mock cells was performed; the blots were probed with anti-phospho-Nedd4 antibodies (**C**); infected cells were treated with I-2 for 36 h, then harvested for analyzing sub-G1 phase using PI staining (**D**). ** *p* value < 0.01; *** *p* value < 0.001 compared with mock-treated infected cells.

To analyze the possible inhibitory mechanism(s) of CW-33 on JEV-induced intracellular Ca^2+^ that linked with the activation of Akt/mTOR, and Jak/STAT signal, phosphoproteomic profiling of infected cells treated with or without CW-33 was determined using enriched phosphopeptides by the TiO2-PDMS plate and quantitative LC-MS/MS (Supplementary Table S1). Figure 8A showed MS/MS spectra for mapping phosphopeptides of protein phosphatase inhibitor 2 (I-2). Protein class analysis of proteins identified using PANTHER classification system indicated that 21.6% (11/51) belonged to the enzyme modulator category, such as protein phosphatase inhibitor 2 (I-2), Rho GTPase-activating protein 35, ARF GTPase-activating protein GIT2, Ras-related GTP-binding protein C, putative 3-phosphoinositide-dependent protein kinase 2, Serine/threonine-protein kinase SIK3, and Serine/threonine-protein kinase N2 (Figure 8B). Western blotting analysis of phosphorylation status of identified proteins like E3 ubiquitin-protein ligase NEDD4-like showed the consistent pattern of protein phosphorylation in treated infected cells with the phosphoproteomic profiling identified by LC-MS/MS (Figure 8C). In addition, protein phosphatase-1 inhibitor-2 (I-2) identified was decreased by nearly 60% in JEV-infected cells, but regained in infected cells post-treatment with CW-33. Moreover, I-2 was used to treat JEV-infected cells for ascertaining the relationship of JEV infection with I-2 (Figure 8D). Interestingly, I-2 at 5 nM significantly reduced the sub-G1 (apoptotic) fraction in JEV-infected cells. The result indicated that CW-33 regulated enzyme modulators like I-2, Rho GTPase-activating protein 35, ARF GTPase-activating protein GIT2, and Ras-related GTP-binding protein, which are involved in the activation of Akt/mTOR and/or Jak/STAT in infected cells.

## 3. Discussion

The study identified antiviral activities of the novel compound CW-33 against JEV infection, including the inhibition of viral CPE, virus-induced apoptosis, virus yield titer, and plaque formation (Figure 1, Figure 2, Figure 3, Figure 4 and Figure 5). CW-33 had a more potently inhibitory effect on the JEV propagation in vitro (IC_50_ = 12.7 to 38.5 μM) compared to the replication of enterovirus A71 in vitro [15]. Antiviral activity of CW-33 also had a similar efficacy to furanonaphthoquinone derivatives against JEV [16]. Moreover, CW-33 significantly reduced the JEV-induced increase of intracellular Ca^2+^ levels that was associated with the recovery of MMP in infected cells (Figure 6). The inhibition of JEV-induced Ca^2+^ overload by CW-33 also correlated with the antagonistic action of CW-33 on JEV-mediated suppression of Akt/mTOR, ERK1/2, and Jak/STAT pathways (Figure 7). Phosohoproteomic analysis revealed CW-33 regulating the protein phosphatase 1/I-2 complex to activate Akt in JEV-infected cells, which was confirmed by the reduction of JEV-induced apoptosis by CW-33 (Figure 8, Supplementary Table S1). Ca^2+^ overload was observed in cells infected with HIV-1 [21], HTLV-1 [22,23], HCV [24,25], HHV-8 [26], and enterovirus [27], where it was seen to increase Ca^2+^-dependent enzymatic processes, promote virus replication, obstruct immune responses, and regulate mitochondria-mediated apoptosis [28]. The inhibition of JEV-induced Ca^2+^ overload by CW-33 could be responsible for reducing the mitochondria-mediated apoptosis of infected cells.

I-2 has been recognized as an inhibitor for protein phosphatase 1, which significantly dephosphorylates Akt in apoptotic cells [29,30]. JEV infection caused the decreased phosphorylation of I-2, Akt, and mTOR, but CW-33 treatment raised the phosphorylation levels of Akt, and mTOR in a dose-dependent manner (Supplementary Table S1, Figure 7). I-2 treatment significantly reduced the apoptosis (Sub-G1 phase) of JEV-infected cells (Figure 8D). The results implied that protein phosphatase 1/I-2 complexes might be involved in the regulation of Akt-dependent anti-apoptosis. Among the proteins upregulated in infected cells by CW-33 (Supplementary Table S1), putative 3-phosphoinositide-dependent protein kinase 2 (PDPK2P) phosphorylates and activates Akt [31]. Moreover, Akt modulates the Ca^2+^ release from ER, reducing Ca^2+^-mediated apoptosis [20]. Therefore, CW-33 treatment triggered the Akt activation in JEV-infected cells, inhibiting the Ca^2+^-mediated apoptosis of JEV-infected cells.

JEV exhibited Type I interferon antagonistic function by blocking JAK/STAT signal via suppression of Tyk2 tyrosine phosphorylation [32,33]. JEV NS5 protein had Type I IFN antagonistic activity to suppress the phosphorylation and nuclear translocation of STAT1 via activating Ca^2+^/calcineurin signaling pathway [34]. CW-33 significantly inhibited the JEV-induced rise of intracellular Ca^2+^, and activated the JAK/STAT signals (Figure 6 and Figure 7). Thus, the inhibitory effect of CW-33 on the decrease of JEV-induced Ca^2+^ overload could be linked with the CW-33-mediated attenuation of Type I interferon antagonistic activities of JEV. Interestingly, RT-PCR assay indicated CW-33 significantly reduced sense and anti-sense JEV RNA genome (Supplementary Figure S1), which implies that CW-33 affected the enzymatic activity of NS5. Thus, CW-33 might directly or indirectly reduce the enzymatic activity of NS5 and might also be related to the decrease of JEV-induced Ca^2+^ overload by CW-33. Quinoline derivatives such as triazolo[4,5-*g*]quinolines, imidazo[4,5-*g*]quinolones, and pyrido[2,3-*g*]quinoxalines have also been demonstrated to exhibit antiviral activity against pathogenic viruses in the Flaviviridae family, including Bovine Viral Diarrhoea virus (BVDV, Pestivirus), Yellow Fever (YFV, Flavivirus), and Hepatitis C virus (HCV, Hepacivirus). Enzymatic assays indicated that these quinoline derivatives had a significant inhibitory effect on RNA-dependent RNA polymerase (RdRp) activities of viral NS5 proteins [35,36].

Phosphoproteomic analysis indicated that CW-33 upregulated Rho GTPase-activating protein 35, ARF GTPase-activating protein GIT2, and Ras-related GTP-binding protein C (Supplementary Table S1), which are Rho GTPases regulating the G-protein-coupled receptor-mediated STAT-dependent gene expression [37]. Recently, IFN-inducible GTPases, such as Myxovirus resistance (Mx) GTPases, were characterized as a potential target participating in the antiviral process [38,39]. IQ-motif containing GTPase activating protein 2 (IQGAP2) was identified as a novel gene involved in IFN antiviral response against HCV infection. IQGAP2 interacts with the RelA (p65) subunit of the NF-κB, and upregulated the expression of IFN stimulated genes (ISG) [40].

## 4. Materials and Methods

### 4.1. Cells and Viruses

BHK-21 cells were used for the amplification of JEV strain T1P1 and plaque assay; human medulloblastoma TE671 cells were applied for testing anti-JEV activities and mechanism(s) of CW-33. Both types of the cells were grown in Minimum essential Medium (MEM), supplemented with 5% fetal bovine serum (FBS), glutamine, pyruvate, and penicillin/streptomycin.

### 4.2. Synthesis of Compound CW-33

Compound CW-33 (ethyl 2-(3′,5′-dimethylanilino)-4-oxo-4,5-dihydrofuran-3-carboxylate) was synthesized and purified by high-performance liquid chromatography, as described in our prior report [15]. The molecular structure of CW-33 was identified confirmed by nuclear magnetic resonance and mass spectrometry, as described in our prior report [15].

### 4.3. MTT Cytotoxicity Test

BHK-21 and TE671 cells (3 × 10^3^ cells/well) in 96-well plates were grown in MEM containing 2% FBS overnight, and then treated with the indicated concentrations of compound CW-33 (2.5, 25, 125, 250, and 500 μM) in quadruplicate. After another 48-h incubation at 37 °C in 5% CO_2_, 10 μL of 5 mg/mL MTT solution was added to each well, and reacted for 4 h. Subsequently, insoluble purple formazan, converted from MTT by dehydrogenase enzymes, was dissolved by DMSO (100 μL/well); yielding absorbance (OD_570–630_) was measured at a wavelength of 570 nm with background subtraction by a reference wavelength of 630 nm using a micro-ELISA reader. The survival rate, indicating the suppressive effects of compound CW-33 on both these types of cells, was calculated as a ratio of OD_570–630_ of treated cells to OD_570–630_ of untreated control cells. Survival rate (%) = (*A*_treated cells_/*A*_control cells_) × 100%. Cytotoxic concentration giving 50% (CC50) was calculated by a computer program (provided by John Spouge, NCBI, NIH, Bethesda, MD, USA).

### 4.4. Cytopathic Effect Inhibition

To examine the antiviral activity of CW-33 against JEV, cytopathic effect and apoptosis of JEV-infected cells in the presence and absence of CW-33 were determined using microscope photograph, and a*nnexin V-FITC*/propidium iodide staining with flow cytometry. BHK-21 and TE671 cells grown in MEM containing 2% FBS were infected with JEV at MOIs of 0.1 and 0.05, respectively. The infected cells were immediately treated with/without CW-33 (2.5, 25, and 125 μM). JEV-induced cytopathicity was observed and photographed under microscope 24, 36, 48, and 72 h post-infection. Mock control, mock infected, and infected and treated cells were harvested, washed by the binding buffer (10 mM HEPES, 140 mM NaCl, 2.5 mM CaCl_2_, pH 7.4), and then stained using the annexin V-FITC/PI solution for 15 min in the dark. At least 10,000 cells per sample were analyzed by flow cytometry with an excitation wavelength of 488 nm and the emission wavelengths at 530 nm for FITC and 620 nm for PI, respectively.

### 4.5. Western Blotting Analysis of Signaling Pathways

To determine the effect of CW-33 on JEV-induced apoptosis and signal pathway activation, TE671 cells were simultaneously infected with JEV at an MOI of 0.05 and treated with/without various concentrations of CW-33. After a 36-h incubation, the lysate of mock, infected, infected/treated, or treated cells was mixed with the SDS-PAGE sample buffer and heated for 10 min. Proteins in the lysates were resolved by SDS-PAGE and transferred to the nitrocellulose membrane. Resultant membranes were blocked with 5% skim milk, then reacted with appropriately diluted antibodies, including antibodies anti-caspase 3 (IMGENEX, San Diego, CA, USA), anti-phospho-Akt Ser473, anti-phospho-mTOR Ser2248, anti-phospho-JAK1 Tyr1034/Tyr1035, anti-phospho-JAK2 Tyr1007/Tyr1008, anti-phospho-Tyk2 Tyr1054/Tyr1055, anti-phospho-STAT1 Ser727 (Cell Signaling, Danvers, MA, USA), and anti-beta actin (NOVUS BIOLOGICALS, Littleton, CO, USA). Immune complexes were detected with horseradish peroxidase-conjugated goat anti-mouse or anti-rabbit IgG antibodies, followed by enhanced chemiluminescence detection (Amersham Pharmacia Biotech, Piscataway, NJ, USA).

### 4.6. Virus Yield Reduction Assays

For examining the anti-JEV activity of CW-33 on virus yields, a JEV titer in cultured supernatant of infected cells treated with/without CW-33 was measured using plaque assay. Serial dilution of cultured supernatant was added into six-well plates of BHK-21 cell monolayer, incubated at 37 °C in 5% CO_2_ incubator for 1 h, and then overlaid with 2 mL MEM medium containing 1.1% methylcellulose for another 72 h of incubation. Finally, the cell monolayer was stained with naphthol blue-black dye; viral plaques were counted and viral yields were determined by the number of viral plaques per mL.

### 4.7. Virucidal and Attachment Assays

Virucidal assay was based on our prior reports [41,42]. The mixture of JEV (10^5^ pfu per reaction) with indicated concentrations of CW-33 was incubated for 60 min at 4 °C, then the 1000-fold dilution of each mixture was added onto BHK-21 cell monolayer in six-well plates for another 1-h incubation at 37 °C in 5% CO_2_, followed by the protocol of plaque assay. In the virus attachment assay, the mixtures of JEV (100 pfu per well) with various concentrations of CW-33 (0, 2.5, 25, 125, or 250 μM) were immediately added onto BHK-21 cell monolayer in the six-well plates at 4 °C for 1 h. After removing the mixture and washing with cold PBS, the cell monolayer was overlaid with an MEM medium containing 1.1% methylcellulose. After a three-day incubation at 37 °C in a 5% CO_2_ incubator, plaques were stained, as described in plaque assay. Residual infectivity and inhibitory concentration showing 50% JEV plaque reduction (IC_50_) were determined, as described in our prior reports [17,18].

### 4.8. Time-of-Addition Assay

To determine the time-of-addition effect of CW-33 on JEV replication, prior, simultaneous, continuous, and post-treatment experiments were performed. In the prior treatment experiment, BHK-21 cells were pre-treated with/without CW-33 (2.5, 25, 50, and 125 μM) 1 h before infection, infected with 100 pfu of JEV at 37 °C in 5% CO_2_ incubator for 1 h, and then washed with phosphate buffer saline (PBS) for removal of unattached viruses and CW-33. Finally, the cells were overlaid with 2 mL of MEM medium containing 1.1% methylcellulose for a 72-h incubation; viral plaques were counted after staining with naphthol blue-black dye. In the post-treatment experiment, CW-33 was added into cells 1 h post-infection and the plaque assay was followed as in the pre-treatment test. In the simultaneous treatment experiment, cells were simultaneously treated with CW-33 and infected with JEV, and then washed with phosphate buffer saline (PBS) for removal of un-attached viruses and CW-33 1 h post-infection, followed by plaque assay. The continuous treatment experiment was similar to the simultaneous treatment test except no removal of JEV and CW-33 in the plaque assay. Inhibitory concentration showing 50% JEV plaque reduction (IC_50_) was determined using data of three independent experiments.

### 4.9. Detecting Intracellular Free Ca^2+^

Mock cells or infected cells with JEV at a MOI of 0.1 were treated with or without indicated concentrations of CW-33, harvested 36 h post infection. After washing twice with PBS, cells were stained with 10 mg/mL Fluo-3/AM (Sigma, Saint Louis, MO, USA) at 37 °C for 30 min in darkroom for analyzing intracellular free Ca^2+^. After last wash with PBS, cells were analyzed by flow cytometry (excitation at 506 nm and emission at 526 nm) (Becton Dickinson FACS Calibur, San Jose, CA, USA).

### 4.10. Mitochondrial Membrane Potential Assay with JC-1 Dye

To monitor mitochondrial health, JEV-infected cells were treated with CW-33 for 24 h, washed with PBS three times, and then stained with 5 μg/mL of membrane-permeant JC-1 dye as the mitochondrial membrane potential probe (Thermo Scientific, San Diego, CA, USA). After 10 min incubation in the dark, the cells were washed with PBS; the cells with the green fluorescence of JC-1 monomer were photographed using fluorescent microscope.

### 4.11. Quantification of Gene Expression Using Real Time RT-PCR

Total RNA was isolated from mock, infected, infected/treated, and treated cells using PureLink Micro-to-Midi Total RNA Purification System Kit (Thermo Scientific, San Diego, CA, USA). The cDNA was synthesized from 1000 ng of total RNA using the oligonucleotide dT primer and SuperScript III reverse transcriptase kit (Invitrogen). To quantitate the gene mRNA expression in response to JEV and/or CW-33, a two-step RT-*PCR* using *SYBR Green* I was used. Oligonucleotide primer pairs were: (1) forward primer 5′-CAACCAGCGGTTGACTTTTT-3′ and reverse primer 5′-ATCCAGGAAGGCAAACTGAA-3′ for human PKR; (2) forward primer 5′-GATGTGCTGCCTGCCTTT-3′ and reverse primer 5′-TTGGGGGTTAGGTTTATAGCTG-3′ for human 2′-5′-OAS; and (3) forward primer 5′-AGCCACATCGCTCAGACAC-3′ and reverse primer 5′-GCCCCAATACGACCAAATCC-3′ for human GAPDH, respectively. Real-time PCR reaction was performed in ABI PRISM 7700 sequence detection system (Thermo Scientific, San Diego, CA, USA), as described in our prior studies [15,42]. Relative mRNA levels of indicated genes were normalized relative to GAPDH mRNA.

### 4.12. Phosphopeptide Analysis of JEV-Infected Cells Treated with or without CW-33

JEV-infected cells treated with or without CW-33 for 24 h were lysed in RIPA (radioimmunoprecipitation assay) lysis buffer; lysates were dissolved in solutions of 18.5 μL of acrylamide (40%, 29:1), 2.5 μL of 10% ammonium persulfate, and 1 μL of TEMED (tetramethylethylenediamine). Lysate proteins in the acrylamide gel were digested with trypsin; peptides were derived from gel via sequential extraction, and then applied for purification of phosphopeptides and nanoLC-MS/MS analysis, as described above in our prior report [43]. Phosphopeptides were enriched using the TiO_2_–polydimethylsiloxane (PDMS) coated plate, resuspended in 0.1% FA solution, and then identified with a nanoflow UPLC system (UltiMate 300 RSLCnano system, Dionex, Amsterdam, The Netherlands) coupled with a captive spray ion source and a Q-TOF mass spectrometer (maXis impact, Bruker, Fremont, CA, USA). LC-MS/MS spectra were transformed to xml files using DataAnalysis (version 4.1, Bruker) for searching using the Swissport database with the MASCOT algorithm (version 2.2.07) [44]. Label-free quantitative proteomics was accomplished by LC-MS replicated runs; the results were processed to exhibit the molecular feature using DataAnalysis 4.1, ProfileAnalysis software 2.0, and ProteinScape 3.0 (Bruker Daltonics, Fremont, CA, USA) using *t*-tests (Bruker Daltonics). Finally, peptides were identified and quantified.

### 4.13. Inhibition of JEV-Induced Apoptosis by Protein Phosphatase 1 Inhibitor-2 (I-2)

JEV-infected TE671 cells at an MOI of 0.5 were treated with protein phosphatase 1 inhibitor-2 (I-2) (New England Biolabs, Ipswich, MA, USA) at 1 and 5 nM for 24 h, and harvested for cell cycle analysis by propidium iodide (PI) staining. Cell cycle assays were performed using flow cytometry with excitation wavelength of 488 nm and emission wavelength of >575 nm. Sub-G1 (apoptotic), G1, S, and G2 cell phases were measured by BD FACSCanto™ system.

### 4.14. Statistical Analysis

The standard error of the mean in each assay was calculated according to three independent experiments. All data were compared using Student’s *t*-test and Scheffe’s test. *p* < 0.05 was considered to be a statistically significant result.

## 5. Conclusions

CW-33, a novel synthetic derivative of the furoquinoline alkaloid, significantly inhibited JEV replication in vitro, reducing the virus yield in different cell lines with IC_50_ values ranging from 12.7 to 38.5 μM. CW-33 activated Akt signal pathway via recovering I-2 and PDPK2P, modulated ER–mitochondria Ca^2+^ transfer in JEV-induced cells, correlating with reducing the apoptosis of infected cells. CW-33 also diminished the Type I interferon antagonistic activity of JEV through the reduction of the Ca^2+^ overload and the activation of Rho GTPases, as associated with the activation of STAT-mediated antiviral responses.

## Supplementary Materials

Supplementary materials can be found at www.mdpi.com/1422-0067/17/9/1386/s1.
