# Supplementary material for: Antiviral Activity of a Novel Compound CW-33 against Japanese Encephalitis Virus through Inhibiting Intracellular Calcium Overload
Source: Int J Mol Sci. 2016 Aug 24;17(9):1386. doi: 10.3390/ijms17091386 (PMC5037666; doi:10.3390/ijms17091386)
Supplement: Supplementary file 1 [file ijms-17-01386-s001.pdf]

Supplementary Materials: Antiviral Activity of a Novel Compound CW-33 against Japanese Encephalitis Virus through Inhibiting Intracellular Calcium Overload

Su-Hua Huang, Jin-Cherng Lien, Chao-Jung Chen, Yu-Ching Liu, Ching-Ying Wang, Chia-Fong Ping, Yu-Fong Lin, An-Cheng Huang and Cheng-Wen Lin

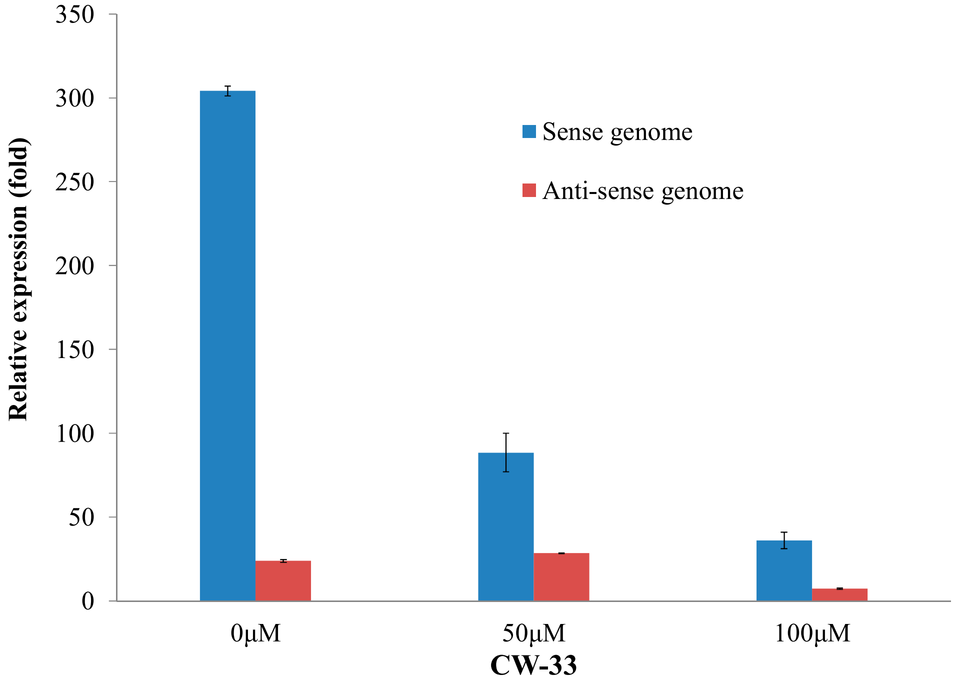

Figure S1. Inhibitory effect of CW-33 on the transcription of JEV replicon. TE671 cells were transfected with the plasmid containing CMV promoter-JEV 5′UTR-EGFP-FMDV 2A-JEV NS proteins 1-5-HDVr-SV40 polyA, and treated with CW-33 1 h post-transfection. Total RNAs were extracted from transfected and treated cells, and reverse transcripted with JEV specific primers. Sense and anti-sense RNA genomes were quantitated using RT-PCR with the NS5 Forward (9724–9743) 5′-TCCACTTCCTCAACGCAATG-3′ and NS5 Reverse (9799–9783) 5′-CAGTCGTGCCAGCCATG-3′. Relative viral sense and anti-sense RNA genomes were measured by quantitative PCR and normalized by GAPDH mRNA, presented as a relative ratio.

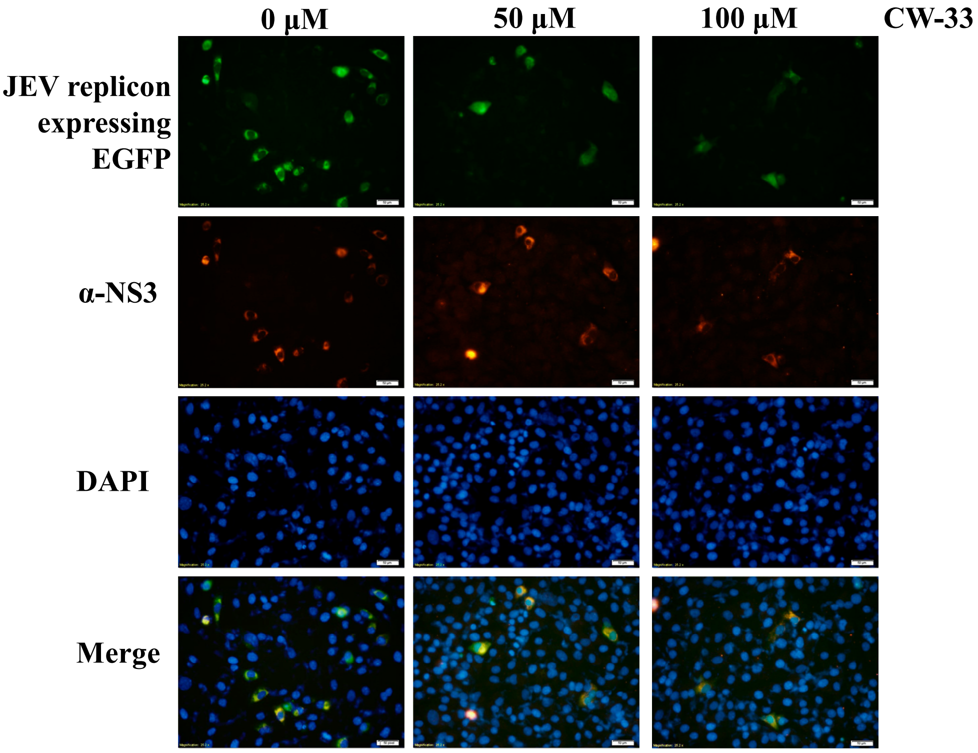

Figure S2. Inhibitory effect of CW-33 on the transcription of the JEV replicon. TE671 cells were transfected with the plasmid containing CMV promoter-JEV 5′UTR-EGFP-FMDV 2A-JEV NS proteins 1-5-HDVr-SV40 polyA, treated with CW-33 1 h post-transfection, and then immunofluorescent staining was performed with anti-JEV NS3 antibodies. Finally, cells were stained with DAPI for 10 min, and imaging analyzed by immunofluorescent microscopy. Scale bars = 50 μm.

Table S1. Protein class analysis of regulated protein-based phosphopeptides identified in JEV-infected cells treated with or without CW-33 by LC-MS/MS.

Protein Class	GI Number	Protein Name	Peptide	Change Ratio of CW-33-Treated Infected Cells to Mock Cells	Change Ratio of Infected Cells to Mock Cells	Sequence Coverage (%)	MASCOT Score
**Calcium-binding protein**							
	6225859	Serine/threonine-protein kinase N2	AS^{p}SLGEIDESSELR	1.07	0.49	1.4	41.4
	74757401	Putative 3-phosphoinositide-dependent protein kinase 2	AN^{p}SFVGTAQYVSPELLTEK	1.15	0.56	4.8	88.8
**Chaperone**							
	17865718	Heat shock protein HSP 90-beta	IEDVG^{p}SDEEDDSGKDKK	1.1	0.67	10.9	442.7
	2495344	Heat shock protein 105 kDa	IE^{p}SPKLER	1.05	0.68	0.9	50.6
**Cytoskeletal protein**							
	116241365	Filamin-A	CSGPGL^{p}SPGMVR	1.13	0.67	0.5	79.1
	2498357	Fascin	NGQLAASVETAGDSELFLMK	1.45	0.82	4.1	71.8
	125962	Prelamin-A/C	SGAQASSTPL^{p}SPTR	1	0.59	2.1	39.3
	13124260	Golgi-specific brefeldin A-resistance guanine nucleotide exchange factor 1	ADAPDAGAQ^{p}SDSELPSYHQNDVSLDR	0.68	0.31	1.4	40.4
	223590110	Kinesin light chain 1	SRE^{p}SLNVDVVK	1.03	0.37	1.9	38.0
**Enzyme modulator**							
	408360250	Rho GTPase-activating protein 35	TSFSVG^{p}SDDELGPIR	0.86	0.3	1	97.0
	17376322	ARF GTPase-activating protein GIT2	QK^{p}SLDSDLSDGPVTVQEFMEVK	0.86	0.37	2.9	46.8
	296452922	TBC1 domain family member 25	QA^{p}SLDGLQQLR	1.13	0.6	1.6	73.4
	34098413	RalA-binding protein 1	TP^{p}SSEEISPTKFPGLYR	0.8	0.41	2.6	46.6
	729856	Protein phosphatase inhibitor 2	IQEQE^{p}S^{p}SGEEDSDLSPEER	0.95	0.38	9.3	38.5
	147644956	Girdin	SS^{p}SQENLLDEVMK	1.42	0.67	0.7	70.8
	13124260	Golgi-specific brefeldin A-resistance guanine nucleotide exchange factor 1	ADAPDAGAQ^{p}SDSELPSYHQNDVSLDR	0.68	0.31	1.4	40.4
	116242760	Ras and Rab interactor 1	^{p}SMSAAFCSLLAPER	1.85	0.89	1.8	64.7
	122065170	Dedicator of cytokinesis protein 7	SLSN^{p}SNPDISGTPTSPDDEVR	0.88	0.53	1	132.2
	294862479	Yorkie homolog	GDSE^{p}TDLEALFNAV^{o}MNPK	1.02	0.44	11.9	489.8
	74752776	Ras-related GTP-binding protein C	MSPNETLFLES^{p}TNK	1.34	0.3	3.5	90.8
**Hydrolase**							
	296452922	TBC1 domain family member 25	QA^{p}SLDGLQQLR	1.13	0.6	1.6	73.4
**Kinase**							
	74757401	Putative 3-phosphoinositide-dependent protein kinase 2	AN^{p}SFVGTAQYVSPELLTEK	1.15	0.56	4.8	88.8
	115502238	Serine/threonine-protein kinase SIK3	RA^{p}SDGGANIQLHAQQLLK	0.89	0.53	1.4	56.8
	6225859	Serine/threonine-protein kinase N2	AS^{p}SLGEIDESSELR	1.07	0.49	1.4	41.4

Table S1. *Cont.*

Protein Class	GI Number	Protein Name	Peptide	Change Ratio of CW-33-Treated Infected Cells to Mock Cells	Change Ratio of Infected Cells to Mock Cells	Sequence Coverage (%)	MASCOT Score
**Ligase**							
	118601083	Acetyl-CoA carboxylase 1	SSM^{p}SGLHLVK	1.04	0.46	1.3	271.5
	73915353	E3 ubiquitin-protein ligase HUWE1	GSGTA^{p}SDDEFENLR	0.79	0.43	0.3	86.2
	73921204	E3 ubiquitin-protein ligase NEDD4-like	SL^{p}SSPTVTLSAPLEGAK	0.78	0.36	1.7	94.7
	50401206	Polymerase delta-interacting protein 2	IFSLSG^{p}TLETVR	1.1	0.63	3.3	42.8
**Membrane traffic protein**							
	147644956	Girdin	SS^{p}SQENLLDEVMK	1.42	0.67	0.7	70.8
	116242760	Ras and Rab interactor 1	^{p}SMSAAFCSLLAPER	1.85	0.89	1.8	64.7
**Nucleic acid binding**							
	417719	40S ribosomal protein S3	LIPDSIGKDIEK	1.13	0.75	10.7	53.6
	74725312	Sister chromatid cohesion protein PDS5 homolog B	AE^{p}SPESSAIESTQSTPQK	0.5	0.21	1.2	42.1
	294862538	Eukaryotic translation initiation factor 4 gamma 1	SF^{p}SKEVEER	0.62	0.22	0.6	61.8
	17376322	ARF GTPase-activating protein GIT2	QK^{p}SLDSDLSDGPVTVQEFMEVK	0.86	0.37	2.9	46.8
**Receptor**							
	115502238	Serine/threonine-protein kinase SIK3	RA^{p}SDGGANIQLHAQQLLK	0.89	0.53	1.4	56.8
**Signaling molecule**							
	13124260	Golgi-specific brefeldin A-resistance guanine nucleotide exchange factor 1	ADAPDAGAQ^{p}SDSELPSYHQNDVSLDR	0.68	0.31	1.4	40.4
**Structural protein**							
	125962	Prelamin-A/C	SGAQASSTPL^{p}SPTR	1	0.59	2.1	39.3
**Transcription factor**							
	294862479	Yorkie homolog	GDSE^{p}TDLEALFNAV^{o}MNPK	1.02	0.44	11.9	489.8
**Transfer/carrier protein**							
	74757401	Putative 3-phosphoinositide-dependent protein kinase 2	AN^{p}SFVGTAQYVSPELLTEK	1.15	0.56	4.8	88.8
	6225859	Serine/threonine-protein kinase N2	AS^{p}SLGEIDESSELR	1.07	0.49	1.4	41.4
	20978413	Collagen type IV alpha-3-binding protein	SS^{p}SMSSIDLVSASDDVHR	1.36	0.78	2.9	57.1
**Transferase**							
	74757401	Putative 3-phosphoinositide-dependent protein kinase 2	AN^{p}SFVGTAQYVSPELLTEK	1.15	0.56	4.8	88.8
	115502238	Serine/threonine-protein kinase SIK3	RA^{p}SDGGANIQLHAQQLLK	0.89	0.53	1.4	56.8
	223590110	Kinesin light chain 1	SRE^{p}SLNVDVVK	1.03	0.37	1.9	38.0
	6225859	Serine/threonine-protein kinase N2	AS^{p}SLGEIDESSELR	1.07	0.49	1.4	41.4
